# Integrated psychological treatment for substance use and co-morbid anxiety or depression vs. treatment for substance use alone. A systematic review of the published literature

**DOI:** 10.1186/1471-244X-9-6

**Published:** 2009-02-20

**Authors:** Morten Hesse

**Affiliations:** 1 University of Aarhus, Centre for Alcohol and Drug Research, Artillerivej 90, 2300 Copenhagen S, Denmark

## Abstract

**Background:**

There is an increasing consensus in favour of integrated treatment of substance use disorders and co-morbid conditions, such as depression or anxiety. However, up till now no systematic reviews have been published.

**Methods:**

Based on a systematic search of MedLine and PsychInfo, 9 trials of integrated treatment for depression or anxiety plus substance use disorder were identified. Where possible, meta-analyses were carried out, using random effects models.

**Results:**

Meta-analyses were carried out for integrated treatment for depression and substance use disorders on a number of outcomes. No meta-analysis could be carried out for integrated treatment for anxiety and substance use disorders, due to multivariate reporting of outcomes in original articles. Integrated treatment for depression and substance abuse produced significant effects on percent days abstinent at follow-up. Differences in retention and symptoms were non-significant, but favoured the experimental condition. For studies of integrated treatment for co-morbid anxiety disorders and substance use disorders, no meta-analysis could be carried out. Several studies of integrated treatment for anxiety and substance use disorders reported that patients assigned to substance use treatment only fared better.

**Conclusion:**

Psychotherapeutic treatment for co-morbid depression and substance use disorders is a promising approach, but is not sufficiently empirically supported at this point. Psychotherapeutic treatment for co-morbid anxiety and substance use disorders is not empirically supported. There is a need for more trials to replicate the findings from studies of integrated treatment for depression and substance use disorders, and for the development of new treatment options for co-morbid anxiety and substance use disorders.

## Background

The prevalence of other psychiatric diagnoses among those with substance-related disorders is notable [1,2]. Around 25% of people in the community in the United States with alcohol dependence and around 50% of people with drug dependence have co-morbid depression [2]. Anxiety disorders can be diagnosed in around 25% of alcohol dependent people and 43% of drug dependent people [2]. Jané-Llopis and Matytsina reviewed surveys from around the world, and found that studies have shown converging evidence for high co-morbidity, recommending that integrated services should be available for patients with co-morbid psychiatric conditions and substance use disorders [1].

Co-morbid psychiatric diagnoses accompanying alcohol addiction, such as severe cases of anxiety or depression, may have a negative impact on quality of life, and on functioning and ability to respond to treatment [3,4].

However, little is known about how to effectively treat these common co-morbidities in substance abusers. Tiet and Mausbach reviewed the literature on integrated treatment, and found that few studies have assessed the effectiveness of integrated treatments for substance use disorders with co-morbid disorders [5].

There is currently some evidence that medications can be effective for co-morbid depression and substance use disorders [6,7].

Among non-substance using patients with anxiety and depression, there is a substantial amount of evidence supporting the efficacy of psychotherapies. For instance, there is robust evidence that cognitive-behavioural therapies can be effective for anxiety disorders [8], and short-term psychodynamic therapy as well as cognitive-behavioural therapy has been shown to be effective for mood disorders such as depression and dysthymia [9].

There is less evidence that non-somatic treatments such as psychotherapy or behaviour therapy is effective for co-morbid depression and substance use disorders (SUD), and there are no empirically supported treatments for co-morbid anxiety and substance use disorders.

In principle, psychotherapies could be useful in at least three ways: psychotherapies could alleviate the symptoms of depression or anxiety, thereby improving the patients' quality of life. They could help patients understand their co-morbidity better, so that the patients were able to understand the link between psychiatric symptoms and relapse, thereby enhancing better coping with urges and cravings, and thereby directly reduce substance use independently of effects on depression and anxiety. And finally, they could improve both. In this review, both outcomes in terms of substance use and in terms of symptoms are considered. For any review that combines data, it is a great challenge when the measures representing related outcomes vary. The choice of the reviewer may be to exclude a substantial proportion of the literature in order to obtain a homogeneous outcome measure, or to combine various outcomes. Both decisions lead to advantages and disadvantages for the outcomes of the review. Excluding a large number of studies may mean that clinically important information is lost. Combining very different measures may result in the inclusion of measures that lack validity and reliability, thereby leading to the wrong conclusions. However, what is available to the reviewer is not known until the literature search has been conducted. For this reason, there is a degree of compromise involved.

## Methods

A comprehensive search of Medline and PsycINFO was carried out, combining terms reflecting depression 1. ("mood" or "depression" or "depressive"), or 2. ("anxiety") with terms reflecting substance use disorders ("substance abuse" or "substance dependence" or "cocaine" or "heroin" or "cannabis" or "alcohol"), and terms reflecting randomized controlled designs ("randomized" or "control group") and terms reflecting non-somatic interventions ("behavioral therapy" or "psychotherapy" or "psychosocial intervention"). Additional searchers using the same search-words were carried out on the Cochrane register of clinical trials and CINAHL. References of included studies were hand-searched, and registers of clinical trials were searched (Clinical Trials, Current Controlled Trials, controlled-trials.com, National Health Service Research and Development Health Technology Assessment Programme (HTA), National Institute of Health, ClinicalTrials.gov). Additional searches were also carried out using "MDMA" as a keyword, but no further studies were identified.

### Inclusion criteria

All patients in the sample had substance use disorders. Only adults included in the study. Patients in the sample had been selected for depressive or anxiety symptoms or a diagnosis of depression or anxiety. The study compared an integrated non-somatic treatment for both substance use disorders and depression or anxiety with a treatment programme solely focusing on the substance use disorder. The study employed a randomized controlled design. Data at post-treatment and/or at follow-up on retention, psychiatric symptoms or substance use outcomes. Only published studies were included. No language restrictions were used.

### Exclusion criteria

Interventions with a mixture of somatic and non-somatic treatments (i.e. where patients were randomized to a combination of pharmacotherapy and psychotherapy vs. placebo). Note that studies where subjects who were randomized to non-somatic treatments were included, even if they were allowed to receive pharmacotherapy during the trial. Studies were excluded only if the experimental condition differed from the control condition in terms of medication status, or if schizophrenia spectrum disorder or personality disorder required for inclusion into study.

### Data extraction and analysis

Data were extracted by the author alone. In the present review, data were analysed using the following outcome measures:

#### Substance use

As all studies that reported substance use outcomes reported percent days abstinent (PDA), and as PDA is a widely used and well-validated measure of substance use severity, this measure was chosen as the indicator of substance use, and the Weighted Mean Difference (WMD) between the control and experiment group was estimated at the follow-up point most commonly reported (6 months).

#### Interviewer-rated symptoms

As all studies that reported interviewer-rated symptoms used the Hamilton Rating Scale for Depression (HRSD), and as the HRSD is a widely used and well-validated measure of depression, this measure was chosen as the outcome measure for interviewer-rated symptoms, and the WMD between the control and experiment group was estimated at the follow-up points.

#### Completion of treatment

From studies that reported differential retention in treatment, the proportion of patients who completed the experimental and control treatment was extracted.

Meta-analyses were conducted using RevMan 4.1 from Cochrane Collaboration, and are presented in RevMan forest plots. For Hamilton Rating Scales and Percent Days Abstinent, Weighted Mean Differences [WMD] were calculated. For all self-report measures of anxiety and all other substance use outcomes combined, Standardized Mean Differences [SMD] were calculated. For retention in treatment, Odds Ratio [OR] was calculated. For all meta-analyses, random effects models were used, as the studies were heterogeneous both with regard to interventions and types of patients. For all outcomes, the I^2 ^statistic was reported. The I^2 ^represents the degree of heterogeneity and can range from 0 to 1. The group that first presented the I^2 ^statistic tentatively suggested that values of 25%, 50%, and 75% represent low, moderate, and high heterogeneity [10]. The I^2 ^statistic represents the extent of heterogeneity, rather than the statistical significance of heterogeneity.

## Results

In Medline, this search resulted in 118 hits, and in PsycINFO, it resulted in 155 hits. Reviews and titles suggesting non-somatic treatments for co-morbid anxiety or depression and substance use disorders were retrieved. Reference lists of reviews and published studies were examined, and studies reporting randomized controlled trials were included. Searches of Cochrane register of clinical trials and CINAHL did not provide additional studies.

Of these reported articles, ten randomized controlled trials that satisfied the inclusion criteria were identified. Five studies were identified that compared integrated treatments for mood disorders and substance use disorders with treatment only for substance use disorders, and five studies that compared integrated treatment for anxiety and substance use disorders with treatments for only substance use disorders.

### Description of studies

Five randomised studies provided manual-guided treatment for co-morbid depression or depressive symptoms and substance use disorders [11-15]. Details of the studies are provided in table 1 and 2.

**Table 1:** Characteristics of studies integrating treatments for depression and substance use disorders

	Type of participants	Proportion receiving antidepressants	Criterion for depression	Interventions	Outcomes
Bowman et al., 1996 [11]	Male substance abusers in an inpatient treatment program.	Not reported	Elevated symptoms (MMPI or MCMI dysthymia)	Self examination therapy versus current events group	SCL-90-R depressive symptoms post-treatment, retention
Brown et al., 1997 [13]	Alcohol dependent patients recruited from a day partial hospital program	45%	Elevated symptoms (BDI)	Coping with depression versus relaxation training	HRSD and BDI post-treatment, Percent Days Abstinence at follow-up
Brown et al., 2006 [12]	Alcohol dependent patients recruited from a dual diagnosis program	97%	CIDI major depressive disorder	Integrated cognitive behavioural therapy versus Twelve Steps Facilitating therapy	HRSD and Percent Days Abstinence post-treatment and at follow-up, and retention
Daughters et al., 2008 [14]	Inpatients dependent on various substances	10%	Elevated symptoms (BDI)	LETs act versus treatment as usual	HRSD and BDI post-treatment and retention
Markowitz et al, 2008 [15]	Patients recruited through flyers and advertising	0%	SCID Dysthymia	Interpersonal therapy versus brief supportive psychotherapy	HRSD, BDI, and PDA

**Table 2:** Number randomized and followed up in included studies

	Number randomized (experimental/control)	Number followed (experimental/control)
Bowman et al., 1996 [11]	14/14	11/11
Brown et al., 1997 [13]	19/16	18/15
Brown et al., 2006 [12]	48/42	28/18
Daughters et al., 2008 [14]	22/22	20/19
Markowitz et al, 2008 [15]	14/12	8/10

The studies are small, with only 223 patients randomized in total. One study used a rarely studied model called self-examination therapy [11], two studies assigned patients to cognitive-behavioural therapy or control [12,13], one study assigned patients to a mainly behavioural intervention [14], and one study assigned patients to interpersonal psychotherapy vs. placebo [15].

Bowman and colleagues compared Self-examination therapy with an attention placebo treatment for co-morbid depression in patients with substance use disorders and co-morbid depressive symptoms during inpatient treatment [11]. In Self-examination therapy, "people are given a booklet which uses a flow chart format and encourages them to: (a) determine what matters to them, (b) think less negatively about things that do not matter to them, (c) invest their energy in things that are important to them, and (d) accept situations they cannot change." [[11], p. 130]. The authors did not report the number of patients receiving medication.

Richard A. Brown and colleagues compared cognitive therapy for depression with relaxation training as part of a partial hospitalization program for alcohol dependence [12]. The cognitive therapy condition, called the "Coping with depression course", incorporated training in depression-relevant skills such as mood monitoring, pleasant activities, constructive thinking, and social skills. [12]. Nearly half the patients (45%) received medication.

Sandra A. Brown and colleagues compared integrated cognitive therapy for depression and substance dependence with Twelve Steps Facilitating therapy [13]. Patients were recruited from a dual diagnosis clinic, and all patients were diagnosed using a structured interview (the CIDI). Almost all (97%) patients received pharmacotherapy for depression.

Daughters and colleagues provided a brief behavioural activation therapy for patients with co-morbid depression and illicit drug use. The program was called Life Enhancement Treatment for Substance Use (LETS Act) [14]. The treatment ran over 6 sessions, plus optional maintenance sessions, and involved among other things defining life goals, identifying relevant activities, self-monitoring and progressive muscle relaxation.

Finally, Markowitz and colleagues allocated patients with dysthymia and alcohol problems to interpersonal psychotherapy or supportive psychotherapy.

### Meta-analyses

**Figure 1:**
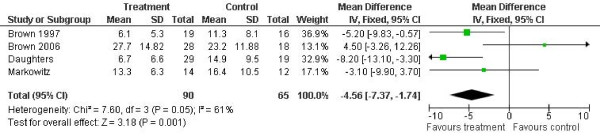
**Hamilton Rating Scale for Depression outcomes**. Notes: SD: Standard deviation. Z is the Zeta statistic for significance of pooled effect size. I^2 ^is the degree of heterogeneity of the effect size. CI: Confidence interval.

**Figure 2:**
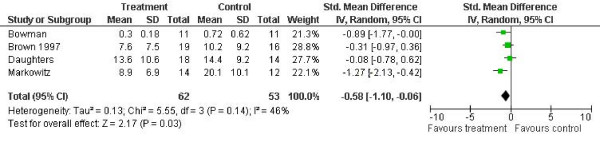
**Self-reported depressive symptoms outcome (SCL-90 or BDI)**. Notes: SD: Standard deviation. Z is the Zeta statistic for significance of pooled effect size. I^2 ^is the degree of heterogeneity of the effect size. CI: Confidence interval.

**Figure 3:**
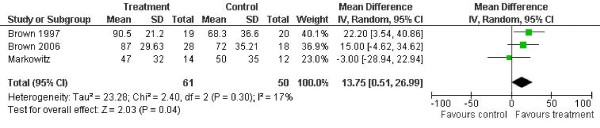
**Percent days abstinent outcomes**. Notes: SD: Standard deviation. Z is the Zeta statistic for significance of pooled effect size. I^2 ^is the degree of heterogeneity of the effect size. CI: Confidence interval.

**Figure 4:**
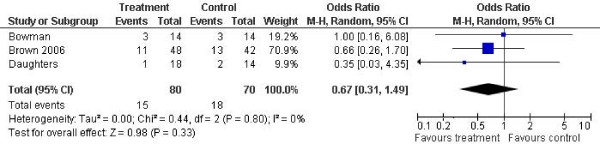
**Drop-out from treatment outcomes**. Notes: SD: Standard deviation. Z is the Zeta statistic for significance of pooled effect size. I^2 ^is the degree of heterogeneity of the effect size. CI: Confidence interval.

In terms of outcomes measured as depressive symptoms, the results of the meta-analyses are shown in figures 1, 2, 3 and 4.

Four studies reported Hamilton Rating Scale for Depression outcomes at post-treatment [HRSD] [12-15]. The combined effect was -4.6 points on the HRSD for experimental condition compared with control (95% confidence interval -7.4 to 1.7). There was significant and moderately high heterogeneity in the outcome (I^2 ^= 0.61, p = 0.05).

Four studies reported self-report questionnaire outcomes for depression [11,12,14,15]. The combined effect was d = -0.58 (95% confidence interval -1.10 to -0.06). Heterogeneity was not significant and low to moderate (I^2 ^= 0.46, p = 0.14).

Three studies reported percent days abstinent after treatment at follow-up [12,13,15]. The combined effect was a reduction of 14.13 in percent days abstinent for the experimental condition (95% confidence interval 2.14 to 26.12, p = 0.02). Heterogeneity was low (I^2 ^= 0.17, p = 0.30).

Three studies reported drop-out from treatment [11,13,14]. The pooled effect size was 0.67 (95% confidence interval 0.31 to 1.49, p = 0.33).

### Integrated treatment for anxiety and substance use disorders versus treatment for substance use disorders alone

Five trials have been published of the treatment of co-morbid anxiety and substance dependence, three targeting alcohol dependence [16-18], and two targeting mixed populations of drug and alcohol abusers [19,20]. All studies have assigned patients to variants of cognitive-behavioural interventions.

As two of the five trials reported only multivariate outcomes, no meta-analysis was conducted [16,17]. Instead, the findings will be reviewed in the following.

Bowen and colleagues randomly assigned inpatients to either alcoholism treatment alone or to alcoholism treatment plus cognitive-behavioural therapy for co-morbid anxiety [16]. The treatment group received 12 hours of CBT for panic disorder in addition to the regular 4-week alcoholism treatment program, and the control group received only the regular program. No treatment group effects were observed on social anxiety or alcohol drinking indices.

Fals-Stewart and Schafer assigned patients in a therapeutic community with obsessive-compulsive disorder (OCD) to a control group, progressive muscle relaxation, or individual behaviour therapy for OCD [19]. Patients assigned to OCD treatment had lower NIMH Obsessive-compulsive scale scores at post-treatment and 12 months follow-up than the patients in the other groups, and a higher proportion of these patients remained abstinent (11 of 19, vs. 11 of 38).

Randall and colleagues randomly assigned patients to alcoholism treatment alone, or to alcoholism treatment plus CBT for social anxiety disorder [17]. The findings indicated that anxiety outcomes were similar, but that alcoholism treatment alone was superior in terms of drinking outcomes. The treatment sessions were longer for the integrated treatment (90 minutes versus 60 minutes).

Schadé and colleagues randomly assigned patients to either treatment for alcoholism alone or treatment for anxiety plus alcoholism in weekly 60-minutes therapy sessions [18]. No significant differences were found for drinking outcomes, but anxiety reductions were significantly higher for the integrated treatment group.

Hien and colleagues assigned patients to either treatment as usual in the community, cognitive relapse prevention or a specialized PTSD program, "Seeking Safety", a short-term, manualized cognitive-behavioural treatment that simultaneously addresses trauma and substance abuse. While both intervention groups fared better than the control group, the results did not favour the Seeking Safety intervention over relapse prevention, with overall slightly better outcomes with relapse prevention [20].

## Discussion

The present meta-analysis showed that integrated psychosocial treatment for depression and substance use disorders is a promising approach for patients with this co-morbidity. The analyses favoured integrated treatment over single-focus treatments for percent days abstinent, depressive symptoms and retention in treatment, although percent days abstinent was the only statistically significant finding, and substantial heterogeneity was observed in several outcomes.

The meta-analyses conducted for integrated non-somatic treatment for co-morbid substance use disorders and depression indicated that while in general, outcomes favoured an integrated treatment, the difference was statistically significant for only one out of four selected outcomes (percent days abstinent at follow-up). Thus, while integrated treatment for depression and substance use disorders are promising, additional studies are needed. Also, given the small sample sizes in the studies, a risk exists that negative studies of similar have gone unpublished. The number of studies that could be included was not sufficient to conduct more formal analyses of publication bias.

### Quality and variability of published studies

A substantial improvement in the reporting of clinical trials is expected with the endorsement of the CONSORT statement [21]. The CONSORT statement involves the reporting of data, including the description of study, methods and outcome. None of the included studies adhered to the CONSORT statement [21], although one recent study did provide the flowchart as recommended in the CONSORT statement [14]. In general, however, the reporting of data was adequate and transparent, and outcomes were measured with adequately validated instruments. The main limitation of the studies was the small sample size of all the studies. A serious threat to internal validity of one study in particular was the high attrition of the study by Sandra Brown and colleagues, where less than half the control subjects were included in the follow-up analyses [13]. This is particularly problematic, as this study is also the single largest trial among all the studies of interventions for co-morbid depressive disorders.

Another key issue concerning the quality of the studies is the criteria for depressive disorders. As can be seen from table 1, the diagnostic criteria vary from elevated scores on self-report questionnaires to CIDI and SCID diagnoses. The studies also varied substantially in terms of setting, types of substances used, and type of interventions. This variability strongly suggests that psychotherapeutic treatments should be considered promising rather than supported for co-morbid substance use disorders and depression.

For anxiety disorders, no meta-analysis could be conducted. However, based on this narrative review there is currently little evidence that offering non-somatic treatment for co-morbid anxiety disorders to patients with substance use disorders will yield any significant benefit; several studies report that outcomes for integrated treatment produced worse results that treatment that focused on substance use disorders alone [17,20]. One possible exception is treatment for co-morbid Obsessive-Compulsive Disorder [19], but this is based on a single, very small trial.

At present, there is a need for more and larger trials in order to study the effectiveness of psychological interventions for co-morbid depression and substance use disorders, and a need to develop new treatment options for co-morbid anxiety and substance use disorders. Some studies are ongoing (Treatment of co-morbid Depression and Substance Abuse in Young People, NCT00232284; Group Therapy for Women Prisoners With co-morbid Substance Use and Depression, NCT00606996; Outpatient Adolescent Treatment for co-morbid Substance Use and Internalizing Disorders, NCT00438685).

## Conclusion

Psychotherapeutic treatment for co-morbid depression and substance use disorders is a promising approach, it is not sufficiently empirically supported at this point. Psychotherapeutic treatment for co-morbid anxiety and substance use disorders is not empirically supported. There is a need for more trials to replicate the findings from studies of integrated treatment for depression and substance use disorders, and for the development of new treatment options for co-morbid anxiety and substance use disorders.

## Competing interests

The author declares that they have no competing interests.

## Authors' contributions

MH planned and carried out the review and wrote the manuscript.

## Pre-publication history

The pre-publication history for this paper can be accessed here:


